# Structure function analysis of *P. falciparum* VAR2CSA

**DOI:** 10.1186/1475-2875-9-S2-I4

**Published:** 2010-10-20

**Authors:** Madeleine Dahlbäck, Lars M Jørgensen, Stig Christoffersen, Morten A Nielsen, Thor G Theander, David E Arnot, Sine Larsen, Ali Salanti

**Affiliations:** 1 Centre for Medical Parasitologyat Department of International Health, Immunology and Microbiology, University of Copenhagen CSS, Øster Farimagsgade 5 A, DK-1014 Copenhagen K, Denmark; 2 Department of Chemistry University of Copenhagen, Universitetsparken 5, 2100 Copenhagen, Denmark

## Background

In pregnant women, *P. falciparum-*infected erythrocytes (IEs) express a unique member of the PfEMP1 family named VAR2CSA, which is associated with the ability of the IEs to adhere to chondroitin sulphate A (CSA) in the placenta [1]. Understanding the mechanism behind this specific CSA interaction is important for the optimal design of a vaccine against placental malaria. CSA-binding of single domains of VAR2CSA has appeared to not reflect the specificity of the native IE binding [2].

## Materials and methods

We have shown that full length recombinant VAR2CSA binds with high affinity to CSA [3]. To define important regions we produced a panel of N and C-terminal truncated VAR2CSA proteins. Proteins were expressed in baculovirus infected insect cells and purified by affinity chromatography followed by size exclusion chromatography. Affinity measurements were done on an Attana biosensor and structural analysis (SAXS) was performed in Grenoble.

## Results

Structural analysis of theVAR2CSA recombinant proteins shows that the protein is structured with a large compact head structure where the minimum CSA binding region is also located.

## Conclusion

The minimum CSA binding region ofVAR2CSA spans several single domains; this is in line with data showing that several single domains can induce adhesion blocking antibodies. It remains to be investigated if the minimum binding region is a better vaccine construct than the proposed single domains.
